# Giant Dermatofibroma: A Rare Presentation as a Large Scaly Plaque

**DOI:** 10.1155/2022/2542404

**Published:** 2022-10-26

**Authors:** T. Iqbal, V. Mudaliar

**Affiliations:** ^1^Department of Dermatology, Leighton Hospital, Mid Cheshire Hospitals NHS Foundation Trust, Crewe, UK; ^2^Department of Pathology, University Hospital of North Midlands NHS Trust, Stoke-on-Trent, UK

## Abstract

A rare form of dermatofibroma (DF) is described in the literature as giant dermatofibroma. Due to the rarity and distinct presentation that can be confused with more sinister skin tumours, these can cause diagnostic uncertainty and require clinicopathologic correlation. Familiarity with this rare presentation of an otherwise common entity is required to prevent unnecessary clinical doubt and excessive interventions. We report a case of giant dermatofibroma on the leg of a 29-year-old healthy male that presented with a 7 cm, nonulcerated pink, brown plaque, adding to the limited literature of less than 30 known cases.

## 1. Introduction

A rare variant of this disease has been described as giant dermatofibroma with less than 30 reported cases. Dermatofibroma (DF), also known as fibrous histiocytoma, is a common benign lesion that is frequently encountered in clinical practice with easily recognisable appearance as pink, brown papules or nodules tethered to the skin causing dimple sign and central pallor with surrounding faint pigmentation on dermoscopy. These are slow growing and mostly solitary asymptomatic lesions up to 1.5 cm in diameter. Multiple lesions can be seen in cases of altered immunity [1].

Giant dermatofibromas are usually larger than 5 cm presenting as polypoidal or ulcerated plaques that have a benign course despite their concerning appearance. Most giant DFs reported hitherto have been found in adult patients over limbs and torso with the exceptions of a paediatric case in China and another case of an ulcerated lesion on the head and neck [2, 3]. Apart from two cases of nonulcerated plaques, all other known giant DFs have presented in the form of ulcerated and pedunculated lesions [2–5].

## 2. Case Report

**Figure 1:**
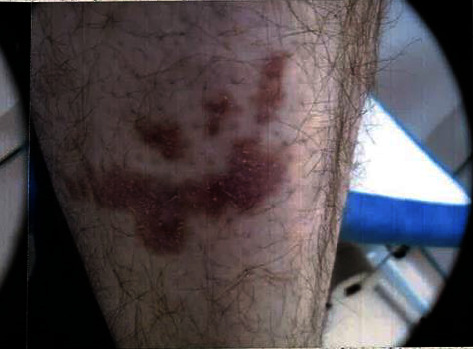
A 7 cm, irregular scaly pink, brown plaque of giant dermatofibroma with satellite lesions on the right shin of a young male. An asymptomatic and slow growing lesion which was nonulcerated.

We report the case of a 29-year-old male who presented with an asymptomatic, pink, brown, scaly plaque of an irregular shape and with some satellite lesions, measuring 7 cm on the right patellar region. This had gradually enlarged over many years. Dermoscopy showed nondiagnostic erythema and some glomerular vessels (Figure 1).

The patient was in good general health with no known systemic conditions or immunosuppression.

**Figure 2:**
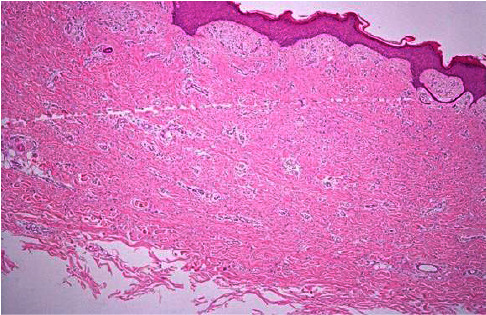
Histopathology of the large plaque with H&E stain at 40*x* magnification. Dermatofibroma with the overlying grenz zone and epidermal hyperplasia.

**Figure 3:**
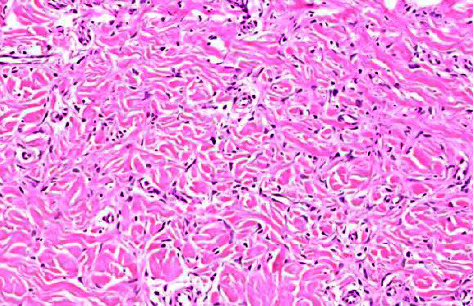
Histopathology H&E stain (magnification × 100). Weaving bland spindle cells around reticular collagen in the dermis.

Histopathology was characteristic of typical DF, showing slightly acanthotic epidermis, and dermal cells with a histiocyte-like elongated appearance of myofibroblasts. There was no cytological atypia or mitotic activity. The lesion extended focally in the subcutis along the septa and was associated with hyalinised collagen and a small number of blood vessels (Figures 2 and 3).

The biopsy was repeated to ensure that histopathology was representative of the lesion, and showed similar results. Further mapping biopsies from the large lesion for an accurate clinicopathologic correlation also supported this finding, confirming the diagnosis of rare giant, atrophic fibrous histiocytoma. We did not find any features of dermatomyofibroma and dermatofibrosarcoma protuberans in histopathology.

Though a benign course was reported in the literature, the patient opted for wide local excision with grafting.

## 3. Discussion

Both neoplastic and reactive pathogeneses have been postulated in the formation of conventional DFs; however, even for giant DFs, no recurrences or metastases have been reported so far after surgical excision, giving these findings a good prognostic outlook. This is supported by this case report. One female patient reported in the literature had muscle ulceration and invasion and developed no metastasis in 5 years after wide resection with myocutaneous flap-plasty [5].

In many cases, giant DFs can mimic sinister lesions such as sarcomas, dermatofibrosarcoma protuberans, desmoplastic melanomas, and nonmelanoma skin cancers, but histopathology reveals storiform arrangement of benign spindle cells. Aneurysmal, heamosiderotic, and xanthomatous histologic variants have been described [2]. Immunostaining is positive for factor VIII A and negative for CD34. In our patient, histopathology showed benign spindle cells ruling out any sinister pathology.

To our knowledge, our report is the third case of a nonulcerated form of this rare entity and a significant addition to the limited literature on giant DFs [4]. Familiarity about this clinical entity among clinicians can lead to avoidance of unnecessary alarm among patients and doctors, as well as need for repeated biopsies and interventions that are prompted by diagnostic uncertainty.
